# Cerebellar metastasis from colorectal cancer: a case report

**DOI:** 10.3389/fonc.2025.1519441

**Published:** 2025-01-24

**Authors:** Carla E. Angulo-Rojo, Luis J. Castillo-Gaxiola, Karen Gaxiola-Gastélum, Alma M. Guadrón-Llanos, Javier A. Magaña-Gómez, Diana L. Baldenebro-Félix

**Affiliations:** ^1^ Laboratorio de Neurociencias, Centro de Investigación Aplicada a la Salud Pública (CIASaP), Facultad de Medicina, Universidad Autónoma de Sinaloa, Culiacán, Sinaloa, Mexico; ^2^ Laboratorio de Diabetes y Comorbilidades, Centro de Investigación Aplicada a la Salud Pública (CIASaP), Facultad de Medicina, Universidad Autónoma de Sinaloa, Culiacán, Sinaloa, Mexico; ^3^ Laboratorio de Nutrición Molecular, Facultad de Ciencias de la Nutrición y Gastronomía, Universidad Autónoma de Sinaloa, Culiacán, Sinaloa, Mexico; ^4^ Programa de Posgrado en Biomedicina Molecular, Facultad de Medicina, Universidad Autónoma de Sinaloa, Culiacán, Sinaloa, Mexico

**Keywords:** case report, colorectal cancer, colonic adenocarcinoma, cerebellar metastasis, tumor markers

## Abstract

**Introduction:**

Colorectal cancer (CRC) is a leading cause of cancer-related mortality worldwide, with adenocarcinoma as the most common subtype. While metastasis typically occurs in the liver, lungs, and peritoneal cavity, metastasis to the brain, particularly the cerebellum, is exceedingly rare.

**Case presentation:**

This report discusses the case of a 50-year-old woman diagnosed with mucinous adenocarcinoma of the descending colon. Over six years, the patient experienced multiple common metastatic sites, including the liver and lungs, before developing a rare cerebellar metastasis. Despite extensive treatment, including surgery and chemotherapy, the disease progressed, ultimately leading to the patient’s demise. This case represents the first documented cerebellar metastasis from CRC in Mexico.

**Conclusion:**

This case highlights the altered metastatic patterns in CRC due to advanced therapies that extend survival. Clinicians should remain vigilant for metastasis to uncommon sites, such as the cerebellum, especially in patients with prolonged survival. Further research is needed to understand the mechanisms underlying such metastatic behavior and optimize treatment strategies.

## 1 Introduction

Colorectal cancer (CRC) is a malignant neoplasm that originates in the colon or rectum, with adenocarcinoma being the most prevalent subtype, accounting for 70-90% of cases (1). CRC is the third most frequently diagnosed cancer in women globally, with an incidence rate of 21.9 per 100,000 individuals. It ranks as the third most common cancer worldwide, contributing to 9.6% of cancer incidence and 9.3% of cancer-related deaths. In 2022 alone, the Global Cancer Observatory reported over 1,926,118 new CRC cases and more than 903,859 associated deaths (2). In Mexico, CRC ranks fourth in incidence, with 207,154 cases, and fourth in mortality, with 96,210 deaths (3). Common metastatic sites in colorectal cancer include the liver, lungs, and peritoneal cavity. Hepatic metastases occur in 30-40% of patients at the time of initial diagnosis and are the leading cause of death. In contrast, pulmonary metastases are found in approximately 15%-20% of patients. Peritoneal metastases are observed in ≤ 10% of patients at diagnosis (synchronous metastases) and in 20%-50% of those with recurrence (metachronous metastases). Brain metastases in colorectal cancer are relatively uncommon compared to other cancers like lung, breast, and renal carcinoma. Studies have indicated that brain metastasis in CRC occurs in 1-3% of cases (4, 5). Despite advances in CRC treatment that have improved patient survival, extended survival durations have been associated with increased metastases in less common sites. Moreover, significant efforts are being made to predict distant metastatic sites and facilitate clinical decision-making (6).

We present a rare case involving a 50-year-old woman diagnosed with colon adenocarcinoma and subsequent metastasis to the left cerebral hemisphere. To our knowledge, this is the first such case reported in Mexico.

## 2 Case presentation

A 50-year-old female patient with no significant medical history presented to the emergency department in November 2011, reporting abdominal distension in the mesogastrium, persistent vomiting, absence of flatus, headache, and vertigo. She was diagnosed with intestinal subocclusion and managed pharmacologically. An abdominal ultrasound (USG) revealed a poorly defined image associated with intestinal loops, leading to a subsequent abdominal computed tomography (CT) scan. The CT scan showed an abnormality in the descending and sigmoid colon, suggestive of a neoplastic process, with no other abnormalities detected. A follow-up colonoscopy on January 17, 2012, identified a tumorous lesion at the junction of the descending and sigmoid colon. A biopsy confirmed the diagnosis of moderately differentiated infiltrating adenocarcinoma.

Preoperative tumor markers were within normal limits except for Carbohydrate Antigen 19-9 (CA19-9) (165 U/ml) and Carcinoembryonic Antigen (CEA) (6.72 ng/ml). A left hemicolectomy was performed, and histopathological examination revealed mucinous adenocarcinoma with signet ring cells in a 20 cm segment of the left colon. The tumor was KRAS-mutated, with metastasis to two peritumoral regional lymph nodes (Dukes stage C), but surgical margins were free of neoplastic involvement. Postoperative CA19-9 levels decreased to 17.28 U/ml. The patient began adjuvant chemotherapy with capecitabine and oxaliplatin (CAPOX regimen) in February 2012, which lasted for six months, after which tumor markers were within normal limits (CA19-9: 7.04 U/ml, CA-125: 8.9 U/ml). The patient remained disease-free for two and a half years.

In July 2014, a routine CT scan revealed chronic calculous cholecystitis, necessitating surgery. Postoperatively, the patient developed icteric syndrome with elevated tumor markers (CA19-9: 191.4 U/ml, CA-125: 78.2 U/ml). Cholangio-MRI identified intrahepatic bile duct dilatation due to obstruction at the common hepatic duct and intra-abdominal fluid accumulation towards the right parietocolic side. A biliodigestive bypass was performed in September 2014 to address bile duct injury from the previous surgery, normalizing liver enzymes and tumor markers. A control colonoscopy in July 2015 showed no abnormalities.

In March 2016, routine laboratory tests detected elevated CA19-9 (53.97 U/ml), but a pelvic-abdominal CT scan showed no significant findings. By May 2017, CA19-9 levels had increased to 541.30 U/ml. Despite normal colonoscopy results, a subsequent oncology consultation in June 2017, prompted by elevated CA19-9 levels and symptoms of skull pain, led to further imaging. CT scans revealed multiple pulmonary metastases and peri-aortic adenomegaly, causing the anterior displacement of the aorta and mass effect on intestinal loops. A bone scan showed an osteoblastic lesion in the right parietal skull, consistent with metastasis. Chemotherapy with FOLFIRINOX plus Bevacizumab was initiated biweekly for six months. CA19-9 levels decreased to 403.1 U/ml after three cycles and 156.2 U/ml after eight cycles (October 2017), indicating disease stabilization. At the end of treatment, CA19-9 levels had further reduced to 82.04 U/ml in January 2018.

In February 2018, a follow-up CT scan revealed periaortic and pericaval adenopathies, nodular pleural wall lesions suggestive of metastasis, liver hypodensities, a hypodense mass in the descending colon (5.8 x 3.6 cm), and a solid hypodense bladder lesion. Due to disease progression and the favorable performance status of the patient (Karnofsky 100, ECOG 0), a third-line treatment with Regorafenib was initiated. The regimen involved three-week cycles with one week of rest over six months. In April 2018, CA19-9 levels rose to 513.3 U/ml, and CEA to 5.16 ng/ml.

**Figure 1:**
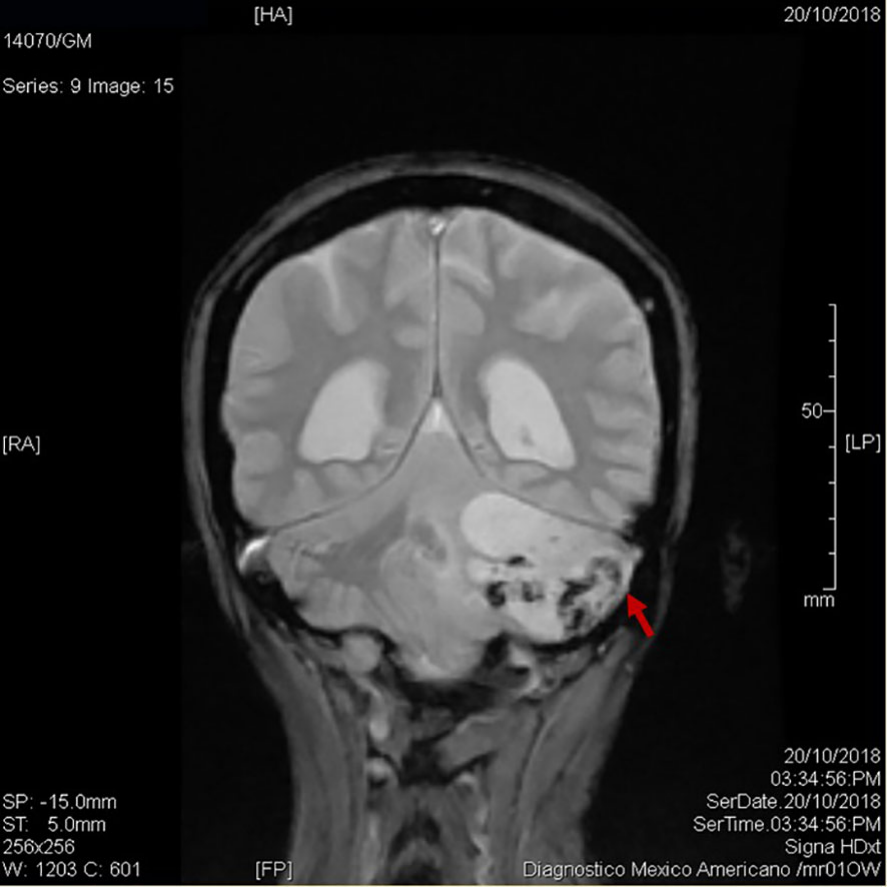
Subsequent MRI of brain cerebellar metastasis. Coronal view: showing an infratentorial mass (36 x 39 x 48 mm) in the left cerebellar lobe (red arrow), leading to tonsillar herniation, compression of the fourth ventricle, and secondary supratentorial ventriculomegaly.

**Figure 2:**
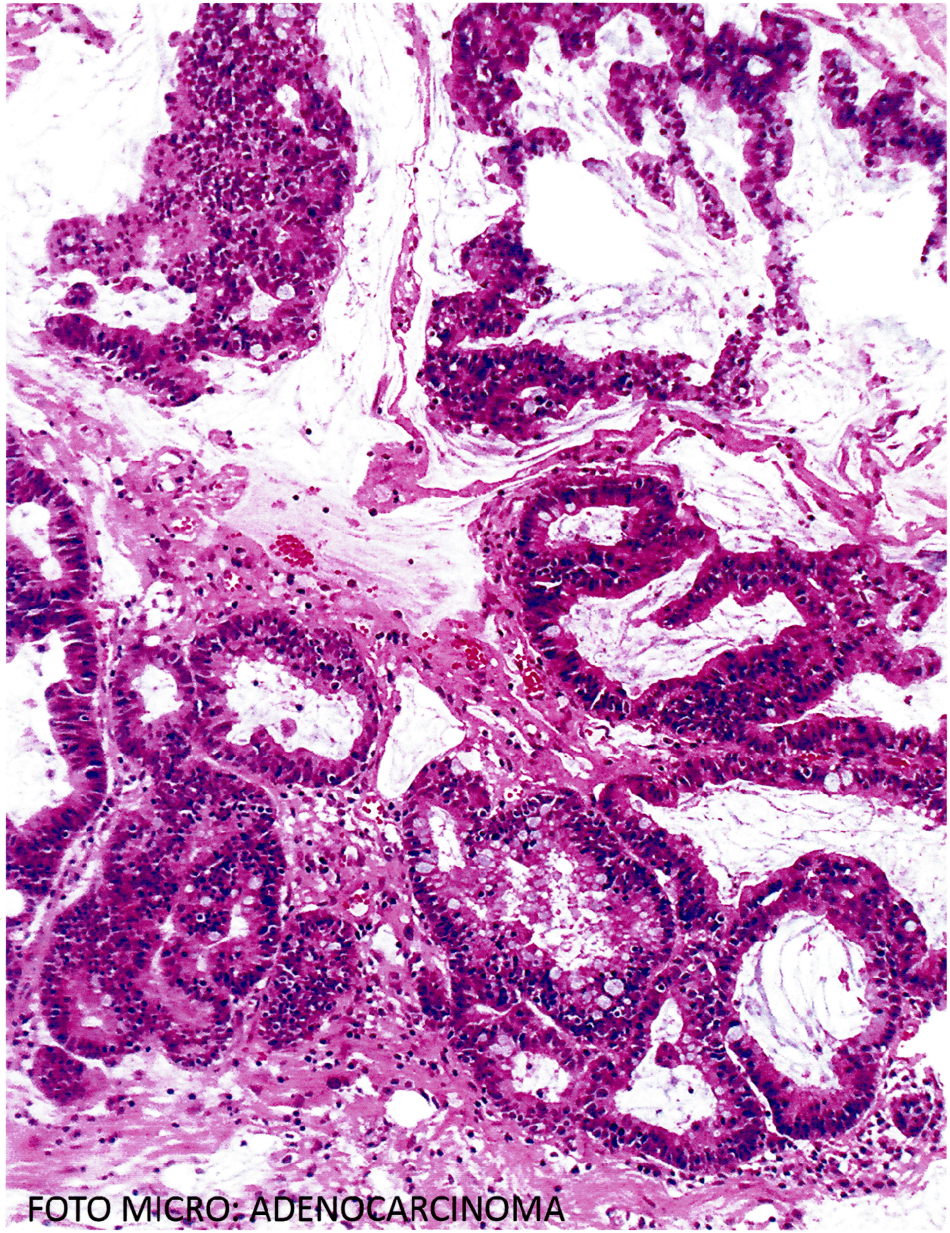
The microscopic analysis showed cerebellum with malignant and invasive epithelial neoplasm with the formation of glandular lumina with cribriform areas lined by cylindrical epithelium and mucinous material (Hematoxylin and eosin staining).

**Figure 3:**
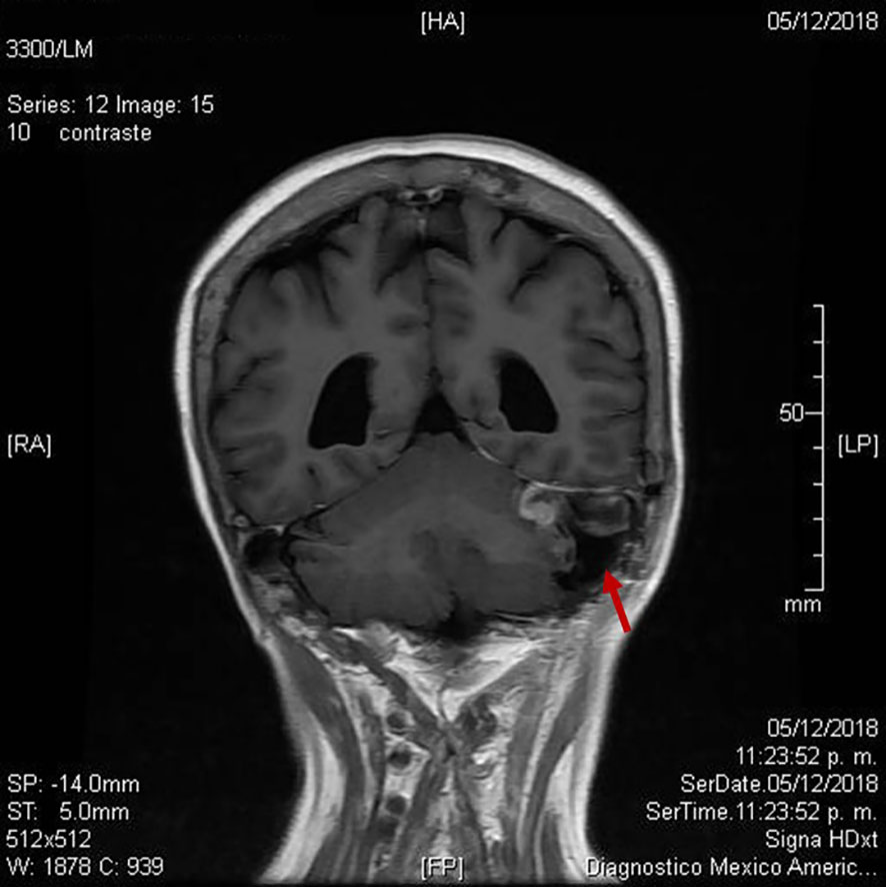
Postoperative magnetic resonance imaging following retrosigmoid craniotomy (red arrow).

**Figure 4:**
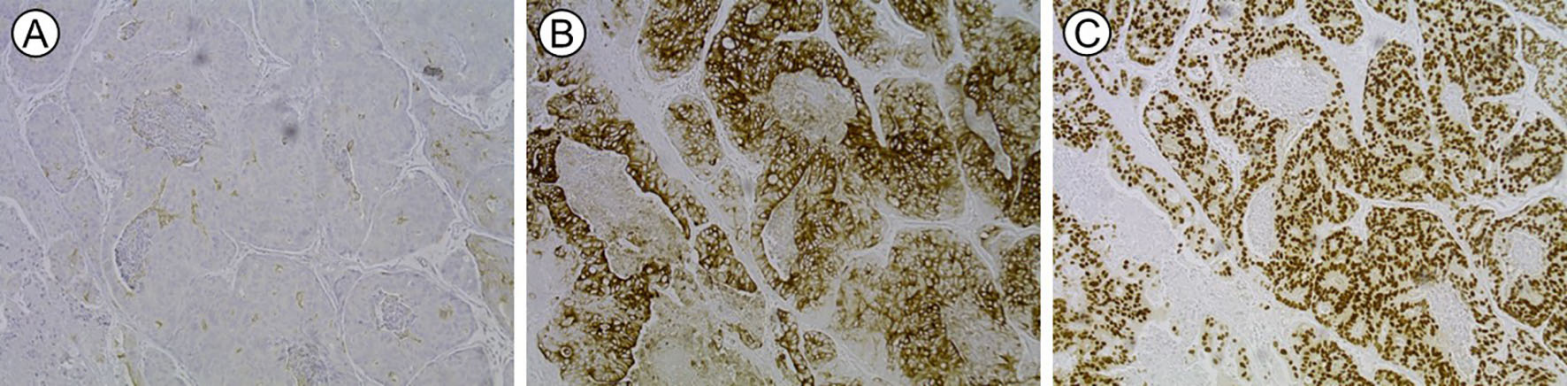
Histopathology confirmed metastatic adenocarcinoma of colonic origin, with positive immunohistochemical staining for **(A)** CK 7 (+), **(B)** CK 20 (3+) and **(C)** CDX 2 (3+).

By July 2018, a cranial CT scan was performed for diagnostic purposes due to persistent headaches, revealing a hypodense mass in the left cerebellum (22 x 26 x 24 mm) with diffuse borders, raising suspicion of cerebellar metastasis. Additionally, multiple lytic skull lesions were identified. Tumor marker levels continued to rise, reaching CA19-9 levels of 1608 U/ml and CEA of 27.62 ng/ml by October 2018. Due to the cerebellar syndrome and elevated biomarkers, a brain MRI was performed, which confirmed cerebellar metastasis. The MRI showed an infratentorial mass (36 x 39 x 48 mm) in the left cerebellar lobe, causing tonsillar herniation, compression of the fourth ventricle, and secondary supratentorial ventriculomegaly (**Figure 1**). On October 29th, 2018, the patient underwent a left retrosigmoid craniotomy with total macroscopic tumor resection. The microscopic analysis showed the cerebellum with malignant and invasive epithelial neoplasm and the formation of glandular lumina with cribriform areas lined by cylindrical epithelium and mucinous material (**Figure 2**). A postoperative MRI scan revealed postsurgical cerebellomalacia changes following tumor resection, with minimal signs of bleeding in the contrast phase. The supratentorial ventricular system appeared normal (**Figure 3**). Days later, histopathology confirmed metastatic adenocarcinoma of colonic origin, with positive CK 7 (+), CK 20 (3+), and CDX 2 (3+) immunohistochemical staining (**Figure 4**). After the surgery, the patient continued with radiotherapy treatment from November 2018 to January 2019. This was done in a private institution, and detailed intervention records are unavailable.

In January 2019, tumor markers reached CA19-9 levels of 48,688 U/ml and CEA of 409.9 ng/ml despite ongoing pharmacological and surgical interventions. On February 8, 2019, a renal ultrasound was ordered because routine blood chemistry tests revealed low serum creatinine concentrations (0.37 mg/dl) and a high blood urea nitrogen/creatinine ratio (52.0), suggesting renal obstruction. The ultrasound revealed a bladder lesion and a decrease in the size of the right kidney. Additionally, generalized ascites was noted. The patient continued to show elevated tumor markers, indicating aggressive disease progression. Despite these efforts, her condition worsened, and she experienced rapid deterioration of her overall health. The patient passed away on February 17, 2019, due to complications associated with the advanced stage of her cancer.

## 3 Discussion

This case reports a rare instance of cerebellar metastasis in a 50-year-old woman with a six-year history of mucinous adenocarcinoma of the descending colon. This is the first documented case of cerebellar metastasis from CRC in Mexico, emphasizing the rarity of this presentation.

Most CRC cases on the left side of the colon (splenic flexure, descending colon, sigmoid colon, rectosigmoid junction) occur in men. In contrast, most cases of CRC on the right side (cecum, ascending colon, hepatic flexure) occur in women (1). This adds to the rarity of our case since our patient had left-sided CRC, which is atypical for a woman. Furthermore, cases follow predictable metastatic patterns, involving the liver, lungs, and peritoneal cavity. However, metastasis to the brain, particularly the cerebellum, is exceedingly rare, occurring in only 1-3% of CRC patients (7). Such cases are often associated with advanced disease and carry a poor prognosis, with survival rates of 3-6 months even with treatment. Notably, this patient presented with mucinous adenocarcinoma, a histological subtype more commonly associated with midgut-origin CRC. Its presence in the descending colon underscores the unusual nature of this case (1, 8). Mucinous adenocarcinomas are known for their aggressive behavior, extensive mucin production, and propensity for peritoneal dissemination, which may have contributed to the patient’s prolonged disease course and atypical metastatic behavior.

Another significant factor was the presence of a KRAS mutation, known to drive tumor aggressiveness, resistance to targeted therapies, and increased metastatic potential. This mutation may have facilitated the invasion and colonization of cerebellar tissue, a rare metastatic site for CRC. The expression of immunohistochemical markers, such as CK 7, CK 20, and CDX 2, confirmed the colonic origin of the cerebellar lesion, further validating the unusual metastatic pattern (9). Furthermore, in this patient, an augment of CA19-9, and CEA concentration was observed through the progression of the case, suggesting that using these markers together enhances diagnostic accuracy for CRC, and probably related metastasis due to both markers often correlated with more aggressive disease characteristics such as lymph node involvement and metastasis (10, 11).

Over six years, the patient developed metastases to common sites (liver, lungs, and peritoneal cavity) before the cerebellum. This suggests that surgical interventions and systemic therapies significantly extended survival beyond the median of 20 to 30 months typically seen in CRC (5, 12). However, this extended survival also allowed time for metastasis to manifest in less common sites, such as the cerebellum. Patients with CRC who develop brain metastases face a significantly poorer prognosis compared to those with metastases in more common organs. Median survival in cases of brain metastases is usually very low, ranging from 3 to 6 months, even with adequate treatment (13). However, median overall survival could range from 0.43 months with best supportive care to 41.1 months with multimodal treatment (surgery/radiation/chemotherapy) (14).

## 4 Conclusion

This case underscores the complexity of metastatic colorectal cancer, particularly in patients who experience prolonged survival due to advances in treatment. Cerebellar metastasis from CRC is considered one of the rarest metastatic occurrences. Advances in systemic therapies and surgical management have significantly improved survival rates for CRC patients. This case emphasizes the importance of continued monitoring for atypical metastatic sites in long-term survivors. The unusual presentation of cerebellar metastasis in a female patient with left-sided mucinous adenocarcinoma highlights the evolving nature of CRC metastasis patterns. Furthermore, it suggests more research to understand the molecular pathways underlying such atypical metastases, focusing on the role of genetic alterations like KRAS mutations. Enhanced understanding may guide more effective surveillance and therapeutic strategies for advanced CRC.

## Data Availability

The original contributions presented in the study are included in the article/supplementary material. Further inquiries can be directed to the corresponding author.
